# The association between an individual’s development of non-communicable diseases and their spouse’s development of the same disease: the Longitudinal Survey of Middle-aged and Elderly Persons

**DOI:** 10.1265/ehpm.24-00294

**Published:** 2025-03-28

**Authors:** Tomohiko Ukai, Takahiro Tabuchi, Hiroyasu Iso

**Affiliations:** 1 Department of Epidemiology and Clinical Research, The Research Institute of Tuberculosis, 3-1-24 Matsuyama, Kiyose, Tokyo 204-8533, Japan; 2 Division of Epidemiology, School of Public Health, Tohoku University Graduate School of Medicine, 2-1 Seiryo-machi, Aoba-ku, Sendai, Miyagi 980-8575, Japan; 3 Cancer Control Center, Osaka International Cancer Institute, 3-1-69 Otemae, Chuo-ku, Osaka 541-8567, Japan; 4 Institute for Global Health Policy Research, National Center for Global Health and Medicine, 1-21-1 Toyama Shinjuku-ku, Tokyo 162-8655, Japan

**Keywords:** Non-communicable disease, Diabetes myelitis, Hypertension, Hypercholesterolemia, Stroke, Heart diseases

## Abstract

**Background:**

Studies have shown that married couples often share similar lifestyles, as well as lifestyle-associated conditions such as diabetes, hypertension, and hyperlipidemia. This study aims to prospectively investigate the association between an individual’s development of a non-communicable disease and the subsequent development of the same condition in their spouse.

**Methods:**

This population-based cohort study utilized 12 waves of annual prospective surveys from 2005 onwards in Japan, with a discrete-time design. A total of 9,417 middle-aged couples (18,834 participants; discrete-time observations = 118,876) were included. Each participant whose spouse had developed one of six conditions was propensity score-matched with five controls whose spouses had not been diagnosed with the condition: diabetes [n = 1374 vs n = 6870], hypertension [n = 2657 vs n = 13285], hypercholesterolemia [n = 3321 vs n = 16605], stroke [n = 567 vs n = 2835], coronary heart disease (CHD) [n = 1093 vs n = 5465] or cancer [n = 923 vs n = 4615]. Using conditional logistic regression, we assessed participants’ development of the same condition within three years following their spouse’s diagnosis.

**Results:**

Participants whose spouses had developed diabetes, hypertension, hypercholesterolemia, or CHD were more likely to develop the same condition within three years. The odds ratios (ORs) and 95% confidence intervals (CIs) were: 1.96 (1.53–2.50), 1.20 (1.06–1.36), 1.63 (1.47–1.81) and 1.43 (1.05–1.95), respectively. No significant associations were observed in stroke [1.69 (0.80–3.58)] or cancer [1.08 (0.75–1.54)].

**Conclusion:**

Spouses of individuals recently diagnosed with certain metabolic conditions are at a higher risk of developing those conditions themselves. These findings may provide valuable guidance for targeting and personalizing chronic disease screening and prevention efforts.

**Supplementary information:**

The online version contains supplementary material available at https://doi.org/10.1265/ehpm.24-00294.

## Introduction

Non-communicable diseases (NCDs) account for the largest number of deaths worldwide [1] and are closely associated with lifestyle-associated risk factors, such as smoking, physical inactivity and obesity [2]. Spouses often exhibit similar behavioral patterns as one another, which could be due to the development of shared habits after marriage or due to assortative mating (i.e., a tendency of individuals to choose a spouse with similar characteristics, such as body mass index, or habits such as smoking) [3, 4]. Such shared lifestyle behaviors can affect partners’ mutual health and may place spouses, both individually and together, at higher risk of NCDs.

Prior literature has suggested when one spouse has a NCD, the other may be more likely to have the same condition themselves. Specifically, cross-sectional studies and their meta-analysis have found that when one spouse had conditions like diabetes, hypertension or hypercholesterolemia, the other was more likely to have the same condition [5–9]. Although these results are complementary they are also conflicting; their findings are constrained by study designs that do not allow inference of causation [10, 11]. For diabetes, prospective cohorts and their systematic review found that spouses of individuals who were newly diagnosed with diabetes developed diabetes at much higher rates than individuals whose spouses did not have the condition [9, 12–14]. In contrast, hypertension and hypercholesterolemia have not been assessed in prospective studies. Furthermore, while a spousal concordance might be expected for cardio-cerebrovascular diseases, considering shared risk factors, this relationship has not yet been explored.

This issue is important, as understanding the relationship between spouses’ health could inform targeted strategies for disease prevention and screening. To address this gap, we analyzed data from the Longitudinal Survey of Middle-aged and Elderly Persons in Japan, examining a broad spectrum of NCDs, including diabetes, hypertension, hypercholesterolemia, coronary heart disease, stroke, and cancer. We hypothesized that a spouse’s development of an NCD would increase the likelihood of the same condition developing in their partner within the subsequent three years.

## Methods

### Study design and population

We used data from the Longitudinal Survey of Middle-aged and Elderly Persons conducted by the Japanese Ministry of Health, Labour and Welfare (MHLW). The purpose of this national survey was to follow middle-aged and elderly men and women to continuously investigate changes in their health, employment, and social activities and to obtain basic data for planning and implementing policies for the elderly [15]. We obtained permission from the MHLW to use the data. The study was reviewed and approved by the Research Ethics Committee of the Osaka International Cancer Institute (no. 1508119060).

The study included Japanese people who were aged 50–59 years as of 31 October 2005; these individuals were selected by a two-stage random sampling procedure. Specifically, the MHLW collected health-related information using self-administered questionnaires from 276,682 households that were randomly sampled from 5280 districts in Japan. From these data, 2515 districts were randomly sampled for the Longitudinal Survey of Middle-aged and Elderly Persons. The sample was not weighted. The survey was conducted every year with the same participants via face-to-face interviews (1st–5th year) or via mail (6th–12th year). In the first survey, 33,815 residents responded, and were followed-up. The number of respondents in the 2nd–12th waves were 31,403, 29,772, 28,492, 27,591, 25,157, 23,672, 22,288, 21,556, 20,680, 20,101 and 19,513. Participants were included for these analyses if they were married and lived with their spouse, and both spouses responded to the questionnaire in the first wave.

### Variables

#### Explanatory variables

The explanatory variables were the spouse’s development of any of the six following conditions: diabetes, hypertension, hypercholesterolemia, CHD, stroke, and cancer. Participants were asked whether they had been diagnosed with any of these conditions by doctors at during the previous year in all 12 waves. When one spouse answered that he or she had not been diagnosed in previous waves but had been newly diagnosed during the most recent year, they were regarded as developing the condition. In the first questionnaire in 2005, participants were asked whether they had been previously diagnosed with any of the conditions (not during the previous year). Therefore, 2006 was the first year when participants could be considered to have newly developed a condition. If participants developed a condition twice, they were excluded the second time.

#### Outcome variables

The outcome of interest for this study was the spouses’ development of diabetes, hypertension, hypercholesterolemia, CHD, stroke, or cancer within 3 years of their partner developing the same condition [12].

#### Covariates

We measured the following potential covariates: age (continuous), sex, education (junior high school, high school, technical school or junior college, or university or higher), household income (0–30,000, 30,001–60,000, 60,001–90 000, or 90,001+ USD), employment status (office worker, manual worker, unknown or unemployed), number of cigarettes smoked each day (0, 1–10 cigarettes/day, 11–20 cigarettes/day, 21–30 cigarettes/day, or 31+ cigarettes/day), alcohol intake (every day, 1–6 times/week, 1–3 times/month, rarely or never) and physical exercise (active and participated in moderate [e.g., walking or jogging] or vigorous [e.g., aerobics or swimming] physical activity at least once a week, or inactive).

### Statistical analysis

**Fig. 1:**
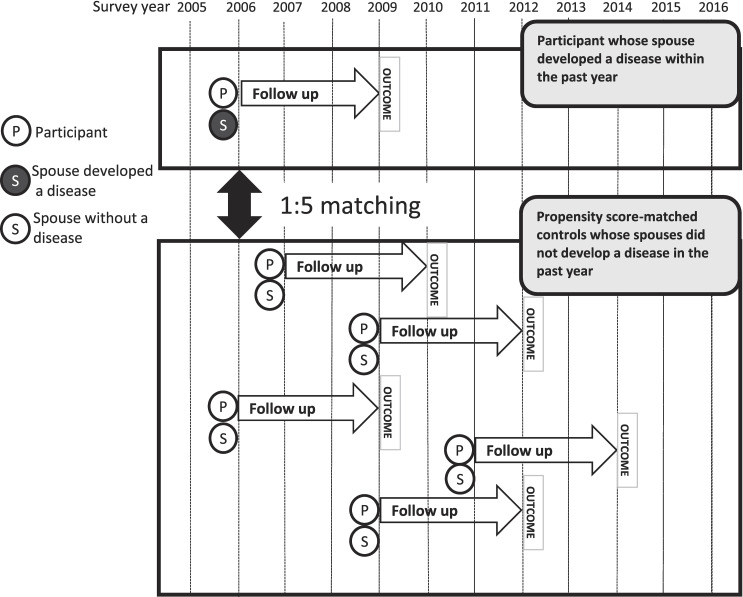
Study design. One participant whose spouse developed the disease, along with five propensity score-matched controls whose spouses were not diagnosed, are followed up for three years.

In each analysis, individuals were included if they had no diagnosis at baseline and had information on their spouse’s status from the previous year. We created discrete-time design modules, in which each wave of participants was treated as an analytical unit. Cases were participants whose spouse had developed a condition within one year. Controls were participants whose spouse did not develop a condition throughout the survey period (Fig. 1).

#### Propensity score matching

Participants’ spouses’ development of condition was not equally scattered, so we conducted a propensity score (PS) analysis to evaluate the association with individual development of the disease. PS matching was used to account for the differences. PS (the probability of spouses’ development of diabetes, hypertension, hypercholesterolemia, CHD, stroke and cancer for each participant ranging from 0–1) was calculated by multivariable logistic regression using the following measured covariates; age, sex, education, household income, employment status, physical activity, smoking status, drinking status. Using this score, we selected five matched couples to enlarge the power without compromising the quality of the matches [12]. We used a nearest neighbor matching algorithm without replacement. To judge the success of each PS matching in terms of creating groups that looked similar on the observed covariates, we used standardized differences; the difference in proportions between the exposed and control groups divided by the standard deviation in the exposed group. Generally, a standard difference of 0.1 indicates a potentially meaningful imbalance (Supplementary 1).

When PS matching created an acceptable balance, incidence proportion and odds ratios (ORs) were calculated. We used a conditional logistic regression model and calculated the incidence of the diseases within 3 years with or without the other spouse’s prior diagnosis [12].

First, we determined the association between one spouse’s prior development of a condition and the onset of the same condition in the other spouse within 3 years of the development. We calculated ORs for each condition (main analysis). Additionally, we examined the association between one spouse’s development of the above-mentioned six conditions and the onset of these six conditions individually in the other spouse (secondary analysis). Therefore, we created 36 (6 × 6) case and control groups. All analyses were performed using SAS version 9.4 software (SAS Institute Inc, Cary, NC).

## Results

Of 33,815 participants in the survey, 18,946 (9,473 couples) were married and living together. In some cases from the first survey in 2005, answers were not obtained from both couples; therefore, 112 (56 couples) were removed, with 18,834 (9,417 couples) remaining for analysis (Table 1). The discrete-time number was 118,876. From that, 1,374 cases in which one spouse developed diabetes in the previous year were matched with 6,870 controls with no diagnosis of diabetes. Similarly, 2,657 cases with hypertension were matched with 13,285 controls without hypertension, 3,321 cases with hypercholesterolemia were matched with 16,605 controls without hypercholesterolemia, 567 cases with stroke were matched with 2,835 controls without stroke, 1093 cases with CHD were matched with 5,465 controls without CHD, and 975 cases with cancer were matched with 4,875 controls without cancer (Table S1).

**Table 1:** Baseline characteristics of participants (9,417 couples) in the first survey in 2005.

	**Husbands (N = 9,417)**		**Wives (N = 9,417)**	

	**n**	**%**	**n**	**%**
Age				
50–54	5675	60.3	3048	32.4
55–59	3742	39.7	6369	67.6
Job				
Not employed	432	4.4	2858	29.4
Manual work	2433	25.0	1293	13.3
Office work	5923	61.0	4303	44.3
Unknown	619	6.4	950	9.8
Missing	10	0.1	13	0.1
Household annual income (USD)				
0–30 000	1944	20.6	1944	20.6
30 001–60 000	4014	42.7	4014	42.7
60 001–90 000	1316	14.0	1316	14.0
90 001–	679	7.2	679	7.2
Missing	1464	15.5	1464	15.5
Education				
Junior high school	1673	17.2	1339	13.8
High school	4194	43.2	4609	47.4
Technical school or junior college	691	7.1	2210	22.8
University (4 years) or more	2137	22.0	618	6.4
Missing	722	7.4	641	6.6
Smoking				
Non-smoker	4875	50.2	8134	83.7
<10 cigarette per day	429	4.4	385	4.0
11–20 cigarette per day	1882	19.4	551	5.7
21–30 cigarette per day	1559	16.0	148	1.5
31– cigarette per day	621	6.4	28	0.3
Missing	51	0.5	171	1.8
Alcohol drinking				
Excessive drinker*	4090	42.1	1002	10.3
Moderate drinker**	4353	44.8	4835	49.8
Non-drinker	1212	12.5	3851	39.6
Missing	59	0.6	26	0.3
Physical activity				
Active†	2391	24.6	2674	27.5
Inactive‡	6513	67.0	6234	64.2
Missing	513	5.3	509	5.2
Comorbid conditions				
History of diabetes	897	9.2	391	4.0
Development of diabetes during follow-up	1259	13.0	707	7.3
History of Hypertension	1868	19.2	1324	13.6
Development of hypertension during follow-up	2671	27.5	1919	19.8
History of hypercholesterolemia	856	8.8	707	7.3
Development of hypercholesterolemia during follow-up	2345	24.1	2296	23.6
History of stroke	160	1.6	70	0.7
Development of stroke during follow-up	525	5.4	280	2.9
History of ischemic heart disease	368	3.8	138	1.4
Development of ischemic heart disease during follow-up	949	9.8	537	5.5
History of cancer	151	1.6	181	1.9
Development of cancer during follow-up	859	8.8	636	6.5

*Excessive drinker: heavy drinker who consumes 15 drinks or more per week for men or 8 drinks or more per week for women, or binge drinker who consumes 5 or more on a single occasion for men or 4 or more drinks on a single occasion for women

†Active: take part in moderate (such as walking or jogging) or vigorous (such as aerobics or swimming) physical activity 4–5 times/week or more

During the three-year follow-up, 3.6% of propensity-score matched participants whose spouse did not develop diabetes, and 6.8% of participants whose spouse had developed diabetes, developed diabetes. The corresponding incidences were 11.5% and 13.5% for hypertension, 11.1% and 17.1% for hypercholesteremia, 1.4% and 2.1% for stroke, 3.4% and 4.8% for CHD and 3.6% and 3.9% for cancer. For diabetes, hypertension hypercholesterolemia, and CHD participants whose spouse had been developed the condition had significantly greater odds of developing the same condition within 3 years compared to participants whose spouse had not been diagnosed with the condition. The ORs (95% confidence interval [CI]) were 1.96 (1.53–2.50), 1.20 (1.06–1.36), 1.63 (1.47–1.81) and 1.43 (1.05–1.95), respectively (Table 2). The same association was not observed for stroke [1.69 (0.80–3.58)] and cancer [1.08 (0.75–1.54)].

**Table 2:** Spouse’s disease linked to the other spouse developing the same disease within three years.

	**Total number**	**Participant’s incidence of ** **diabetes, n (%)**	Odds ratio *

Spouse without prior development of diabetes	6870	246 (3.6)	1 [ref]
Spouse with prior development of diabetes	1374	93 (6.8)	1.96 (1.53–2.50)

	**Total number**	**Participant’s incidence of ** **hypertension, n (%)**	

Spouse without prior development of hypertension	13285	1527 (11.5)	1 [ref]
Spouse with prior development of hypertension	2657	359 (13.5)	1.20 (1.06–1.36)

	**Total number**	**Participant’s incidence of ** **hypercholesterolemia, n (%)**	

Spouse without prior development of hypercholesterolemia	16605	1843 (11.1)	1 [ref]
Spouse with prior development of hypercholesterolemia	3321	567 (17.1)	1.63 (1.47–1.81)

	**Total number**	**Participant’s incidence of ** **stroke, n (%)**	

Spouse without prior development of stroke	2835	40 (1.4)	1 [ref]
Spouse with prior development of stroke	567	12 (2.1)	1.69 (0.80–3.58)

	**Total number**	**Participant’s incidence of ** **heart disease, n (%)**	

Spouse without prior development of CHD	5465	188 (3.4)	1 [ref]
Spouse with prior development of CHD	1093	53 (4.8)	1.43 (1.05–1.95)

	**Total number**	**Participant’s incidence of ** **cancer, n (%)**	

Spouse without prior development of cancer	4875	177 (3.6)	1 [ref]
Spouse with prior development of cancer	975	38 (3.9)	1.08 (0.75–1.54)

CHD, coronary heart disease.

(*) The odds ratio was calculated using conditional logistic regression following propensity score matching.

When a participant’s spouse was diagnosed with diabetes, the participant had high odds of developing only diabetes but not other conditions (Table 3). When a participant’s spouse was diagnosed with hypertension, the participant had high odds of developing hypertension and hypercholesterolemia [1.15 (1.03–1.28)]. When a participant’s spouse was diagnosed with hypercholesterolemia, the participant had high odds of developing hypercholesterolemia, stroke [1.29 (1.00–1.65)] and diabetes [1.20 (1.02–1.41)]. In no case was a spouse’s prior development of a condition associated with the participant’s development of cancer.

**Table 3:** Association between disease development and spouse’s prior disease compared to spouses without prior disease development.

**Spouse’s prior development**	**OR (95% CI) of developing Diabetes**	**OR (95% CI) of developing Hypertension**	**OR (95% CI) of developing Hypercholesterolemia**	**OR (95% CI) of developing Stroke**	**OR (95% CI) of developing ** **CHD**	**OR (95% CI) of developing Cancer**
Diabetes	1.96 (1.53–2.50)	1.17 (0.97–1.42)	1.11 (0.94–1.32)	1.43 (0.94–2.17)	1.18 (0.88–1.59)	0.83 (0.60–1.13)
Hypertension	1.21 (0.98–1.46)	1.20 (1.06–1.36)	1.15 (1.03–1.28)	1.28 (0.96–1.69)	1.16 (0.95–1.40)	1.05 (0.86–1.27)
Hypercholesterolemia	1.20 (1.02–1.41)	0.95 (0.85–1.07)	1.63 (1.47–1.81)	1.29 (1.00–1.65)	1.03 (0.86–1.23)	1.16 (0.98–1.39)
Stroke	1.38 (0.89–2.14)	1.16 (0.85–1.58)	1.51 (1.15–1.96)	1.69 (0.80–3.58)	1.61 (1.02–2.55)	1.47 (0.95–2.26)
CHD	0.90 (0.64–1.27)	1.13 (0.90–1.41)	1.24 (1.02–1.50)	2.13 (1.39–3.25)	1.43 (1.05–1.95)	0.97 (0.68–1.36)
Cancer	1.19 (0.85–1.66)	1.11 (0.88–1.40)	1.16 (0.94–1.43)	1.05 (0.63–1.75)	1.12 (0.79–1.59)	1.08 (0.75–1.54)

OR, odds ratio; CI, confidence interval; CHD, coronary heart disease.

The odds ratio was calculated using conditional logistic regression following propensity score matching.

## Discussion

This prospective cohort study of couples showed that when an individual developed diabetes, hypertension, hypercholesterolemia, or CHD, their spouse was more likely to develop the same condition within 3 years. Previous cohort studies and meta-analyses have shown that similar associations were observed only in diabetes [12]. Our research extended the evidence showing that this was not only the case for diabetes [OR 1.96 (1.53–2.50), incidence proportion 6.8% (case) and 3.6% (control)], but also hypertension [1.20 (1.06–1.36), 13.5% and 11.5%], hypercholesterolemia [1.63 (1.47–1.81), 17.1% and 11.1%] and CHD [1.43 (1.05–1.95), 4.8% and 3.4%] are more likely to occur in spouses of already diagnosed counterparts.

Individuals who live together are likely to engage in many of the same activities, such as eating, physical activity, watching television and smoking, and also experience the same environmental factors [16–18]. Because couples generally have different genetic makeups, our results indicated that shared environmental and behavioral factors could account for the development of diseases. The significant concordance in diabetes, hypertension and hypercholesterolemia suggested that the environment shared by couples had an important role in development of disease. A consequent association for CHD, for which diabetes, hypertension and hypercholesterolemia are risk factors, was also found [19]. However, no such association was found in the development of cancer, a condition that is influenced by both genes and environment differently, depending on the cancer site [20, 21]. Given that cancer was less attributable to lifestyle compared to other NCDs, the lack of association between an individual’s development of cancer and their spouse’s concurrent development is consistent with expectations.

Awareness of an increased risk of developing the same condition as one’s spouse may encourage individuals to adopt preventive behaviors and engage in regular screenings. For public health personnel, a couple-centered approach for spouses, where one spouse has been diagnosed with a noncommunicable disease, could enhance prevention efforts. Our findings also suggest that physicians should consider not only patients’ medical histories but also those of their spouses to comprehensively assess health risks. For instance, if a patient’s spouse has diabetes, the physician can highlight the patient’s elevated risk and recommend focused preventive measures, such as intensified blood glucose monitoring.

Our results further revealed that when a spouse developed a specific condition, the other was more likely to develop the same condition rather than a different one. Although not statistically significant, the odds ratios for spouses both having diabetes, hypertension, or high cholesterol were higher than for other conditions. This suggests that shared risk may stem not only from similar lifestyle habits but also from specific disease-related factors.

While lifestyle changes prompted by a spouse’s health status—such as adjustments to diet or physical activity—can support overall health, these general changes may have limitations. Effective prevention and management may require strategies tailored to each condition’s unique risk factors. Specifically, preventing diabetes, hypertension, and high cholesterol may benefit from individualized approaches that consider the distinct causes and environmental factors linked to each disease.

### Limitations

This study has several limitations. First, participants with a newly-diagnosed spouse may have been more aware of the early symptoms of a particular condition, and this could have made them more likely to consult their physician and be screened. We adjusted for whether participants had health checkups, but there still may have been overestimation. Second, diagnosis of a condition was self-reported by the participants and this may have caused misclassification. Third, the participants were all selected from Japanese opposite-sex couples, aged 50–59 years in 2005 (1st wave), and the results may not be applicable to other populations or other age groups. Fourth, the rate of missing outcome data was 1%–2%. Couples who had separated or in which one spouse had died or dropped out of the study during the study interval were excluded. This may have resulted in an underestimation of the observed associations because couples who dropped out may have been more likely to develop the diseases. Finally, some of the important covariates, including calory intake, may be missed, or there are residual confounders.

## Conclusions

We considered six conditions and found that if a spouse was diagnosed with diabetes, hypertension hypercholesterolemia or CHD, the other spouse, who might share the same risk factors, had a higher chance of developing the same condition. This finding suggests a need for increased attention to the spouses of individuals who are diagnosed with one of the above-mentioned conditions.

## Abbreviations

NCD: non-communicable disease
CHD: coronary heart disease
OR: odds ratio
CI: confidence interval

## Supplementary information

**Additional file 1:** **Table S1.** Comprison of cases and controls (before and after propensity score matching).

## Declarations

### Data availability

Data cannot be shared for privacy or ethical reasons.

## References

[1] Global Health Estimates: Life expectancy and leading causes of death and disability [Internet]. [cited 2022 Sep 6]. Available from: https://www.who.int/data/gho/data/themes/mortality-and-global-health-estimates.

[2] Nyberg ST, Batty GD, Pentti J, Virtanen M, Alfredsson L, Fransson EI, . Obesity and loss of disease-free years owing to major non-communicable diseases: a multicohort study. The Lancet Public Health. 2018;3(10):e490–7. 30177479 10.1016/S2468-2667(18)30139-7 PMC6178874

[3] Sackett DL, Anderson GD, Milner R, Feinleib M, Kannel WB. Concordance for coronary risk factors among spouses. Circulation. 1975 Oct;52(4):589–95. 1080450 10.1161/01.cir.52.4.589

[4] Ukai T, Tabuchi T, Iso H. The impact of spousal behavior changes on smoking, drinking and physical activity: The longitudinal survey of middle-aged and elderly persons in Japan. Prev Med. 2022 Nov;164:107293. 36208818 10.1016/j.ypmed.2022.107293

[5] Di Castelnuovo A, Quacquaruccio G, Donati MB, De Gaetano G, Iacoviello L. Spousal concordance for major coronary risk factors: A systematic review and meta-analysis. Am J Epidemiol. 2009;169(1):1–8. 18845552 10.1093/aje/kwn234

[6] Wang Z, Ji W, Song Y, Li J, Shen Y, Zheng H, . Spousal concordance for hypertension: A meta-analysis of observational studies. J Clin Hypertens. 2017;19(11):1088–95. 10.1111/jch.13084 PMC8030758 28856830

[7] Leong A, Rahme E, Dasgupta K. Spousal diabetes as a diabetes risk factor: a systematic review and meta-analysis. BMC Med. 2014;12:12. 24460622 10.1186/1741-7015-12-12 PMC3900990

[8] Varghese JS, Lu P, Choi D, Kobayashi LC, Ali MK, Patel SA, . Spousal concordance of hypertension among middle-aged and older heterosexual couples around the world: Evidence from studies of Aging in the United States, England, China, and India. J Am Heart Assoc. 2023 Dec 19;12(24):e030765. 38054385 10.1161/JAHA.123.030765 PMC10863781

[9] Appiah D, Schreiner PJ, Selvin E, Demerath EW, Pankow JS. Spousal diabetes status as a risk factor for incident type 2 diabetes: a prospective cohort study and meta-analysis. Acta Diabetol. 2019 Jun;56(6):619–29. 30888538 10.1007/s00592-019-01311-y PMC6520150

[10] Retnakaran R, Wen SW, Tan H, Zhou S, Ye C, Shen M, . Spousal Concordance of Cardiovascular Risk Factors in Newly Married Couples in China. JAMA Netw Open. 2021 Dec 1;4(12):e2140578. 34935919 10.1001/jamanetworkopen.2021.40578 PMC8696567

[11] Watanabe T, Sugiyama T, Takahashi H, Noguchi H, Tamiya N. Concordance of hypertension, diabetes and dyslipidaemia in married couples: cross-sectional study using nationwide survey data in Japan. BMJ Open. 2020 Jul 28;10(7):e036281. 10.1136/bmjopen-2019-036281 PMC7389765 32723739

[12] Cunningham SA, Adams SR, Schmittdiel JA, Ali MK. Incidence of diabetes after a partner’s diagnosis. Prev Med. 2017 Dec;105:52–7. 28823754 10.1016/j.ypmed.2017.08.020 PMC5673498

[13] Nielsen J, Hulman A, Witte DR. Spousal cardiometabolic risk factors and incidence of type 2 diabetes: a prospective analysis from the English Longitudinal Study of Ageing. Diabetologia. 2018 Jul;61(7):1572–80. 29520580 10.1007/s00125-018-4587-1

[14] Hemminki K, Li X, Sundquist K, Sundquist J. Familial risks for type 2 diabetes in Sweden. Diabetes Care. 2010 Feb;33(2):293–7. 19903751 10.2337/dc09-0947 PMC2809269

[15] Ministry of Health, Labour and Welfare. Longitudinal survey of middle-aged and elderly persons [Internet]. [cited 2022 Aug 3]. Available from: https://www.mhlw.go.jp/toukei/list/29-6a.html.

[16] Wilson SE. The health capital of families: an investigation of the inter-spousal correlation in health status. Soc Sci Med. 2002 Oct;55(7):1157–72. 12365528 10.1016/s0277-9536(01)00253-2

[17] Sonneville KR, Rifas-Shiman SL, Kleinman KP, Gortmaker SL, Gillman MW, Taveras EM. Associations of obesogenic behaviors in mothers and obese children participating in a randomized trial. Obesity. 2012 Jul 21;20(7):1449–54. 22349735 10.1038/oby.2012.43 PMC3835375

[18] Macken LC, Yates B, Blancher S. Concordance of risk factors in female spouses of male patients with coronary heart disease. J Cardiopulm Rehabil. 20(6):361–8. 11144042 10.1097/00008483-200011000-00005

[19] Boehme AK, Esenwa C, Elkind MSV. Stroke risk factors, genetics, and prevention [Internet]. Circ Res. 2017;120:472–95. doi: 10.1161/CIRCRESAHA.116.308398. 28154098 PMC5321635

[20] Tamimi RM, Spiegelman D, Smith-Warner SA, Wang M, Pazaris M, Willett WC, . Population attributable risk of modifiable and nonmodifiable breast cancer risk factors in postmenopausal breast cancer. Am J Epidemiol. 2016;184(12):884–93. 27923781 10.1093/aje/kww145 PMC5161087

[21] Stein CJ, Colditz GA. Modifiable risk factors for cancer [Internet]. Br J Cancer. 2004;90:299–303. doi: 10.1038/sj.bjc.6601509. 14735167 PMC2410150

